# Effect of hyperbaric oxygen therapy (HBO) on implant-associated osteitis in a femur fracture model in mice

**DOI:** 10.1371/journal.pone.0191594

**Published:** 2018-01-29

**Authors:** Carina Büren, Tim Lögters, Lisa Oezel, Golnessa Rommelfanger, Armin Olaf Scholz, Joachim Windolf, Ceylan Daniela Windolf

**Affiliations:** Department for Trauma- and Hand Surgery, Medical Faculty, Heinrich-Heine-University, Düsseldorf Moorenstraße 5, Düsseldorf, Germany; CCAC, UNITED STATES

## Abstract

Hyperbaric oxygen therapy (HBO) is applied very successfully in treatment of various diseases such as chronic wounds. It has been already suggested as adjunctive treatment option for osteitis by immune- and fracture modulating effects. This study evaluates the importance of HBO in an early implant-associated localized osteitis caused by *Staphylococcus aureus* (SA) compared to the standard therapy. In a standardized murine model the left femur of 120 BALB/c mice were osteotomized and fixed by a titanium locking plate. Osteitis has been induced with a defined amount of SA into the fracture gap. Debridément and lavages were progressed on day 7, 14, 28 and 56 to determine the local bacterial growth and the immune reaction. Hyperbaric oxygen (2 ATA, 90%) was applied for 90 minutes on day 7 to 21 for those mice allocated to HBO therapy. To evaluate the effect of HBO therapy the following groups were analyzed: Two sham-groups (12 mice / group) with and without HBO therapy, two osteotomy groups (24 mice / group) with plate osteosynthesis of the femur with and without HBO therapy, and two osteotomy SA infection groups (24 mice / group) with and without HBO therapy. Fracture healing was also quantified on day 7, 14, 28 and 56 by a.p. x-ray and bone healing markers from blood samples. Progression of infection was assessed by estimation of colony-forming units (CFU) and immune response was analyzed by determination of polymorphonuclear neutrophils (PMN), Interleukin (IL) - 6, and the circulating free DNA (cfDNA) in lavage samples. Osteitis induced significantly higher IL-6, cfDNA- and PMN-levels in the lavage samples (on day 7 and 14, each p < 0.05). HBO-therapy did not have a significant influence on the CFU and immune response compared to the standard therapy (each p > 0.05). At the same time HBO-therapy was associated with a delayed bone healing assessed by x-ray radiography and a higher rate of non-union until day 28. In conclusion, osteitis led to significantly higher bacterial count and infection parameters. HBO-therapy neither had a beneficial influence on local infection nor on immune response or fracture healing compared to the standard therapy in an osteitis mouse model.

## Introduction

Induction of anti-sepsis and evolution of perioperative standards as well as surgical techniques have significantly reduced the risk of incorporation of bacteria during surgery. Despite these advances, the overall incidence of early infections after a fracture stabilization ranges between 0.1% and 1.7% [1]. In open fractures incidence of infection significantly increases from 2.7% up to 43%, depending on the degree of soft tissue damage and fracture region [1]. Thus, perioperative infections are one of the major challenges in orthopedic and trauma surgery. Some authors designate perioperative infections involving the entire bone including its cortex as “osteitis” and distinguish those infections from infections of the bone marrow (e.g. by hematogenous dissemination) designated as “osteomyelitis” [2,3]. Perioperative infection can result in an osteitis related to a direct contamination of bacteria in open fractures or / and bone stabilization with orthopedic implants. Several infection predisposing factors were identified including the degree of the primary or secondary local tissue damage and systemic host factors such as underlying diseases like diabetes and local vascularity [4,5]. Moreover, the presence of foreign surfaces like plates or prosthesis significantly increases the risk for the development of an infection [6]. In principle, various types of bacteria may cause perioperative osteitis. *Staphylococcus aureus* (SA) and *Staphylococcus epidermidis* (SE) are most commonly responsible for osteitis in pyogenic and medical-device associated osteitis [7,8]. Radical surgical debridement and antibiotic therapy are the main treatment columns of early implant-associated infections. However, bacteria have developed several mechanisms, which allow for growth and evasion of host defense. SA forms a biofilm on foreign surfaces. This biofilm consists of a hydrated matrix of extracellular components including several proteins, in which a multilayer cell cluster of sessile bacteria is embedded [6,8]. Biofilms protect bacteria from the host´s defenses and are resistant against most antibiotics, both enabling SA to cause a chronic infection after fracture stabilization. Biofilm production allows SA to escape from host defense. Next to these mechanisms, SA was considered to directly invade host cells (in particular immune cells such as macrophages and neutrophils) and therefore to undermine the host´s defense [7,9]. Furthermore, fracture healing is impeded by infection progress as well as by a frustrating activation of the innate immune system [10]. Activation of polymorphonuclear neutrophils represents an important mechanism of bacterial defense. However, the release of high levels of potentially cytotoxic molecules like proteases has negative effects on fracture healing [11]. Last, SA develop resistance against antibiotic therapy which poses a serious clinical obstacle to the treatment of osteitis [7]. Despite a radical surgical debridement and antibiotic therapy, recovery from biofilm-associated infections frequently necessitates complete implant removal. Newly developed therapeutic approaches intend to destroy or prevent biofilm formation on implant surfaces. Although with promising results, these approaches have not resulted in a breakthrough regarding the therapy of implant-associated infection [7,12–14]. Moreover, the fracture healing process itself is not addressed by these strategies.

Hyperbaric oxygen therapy (HBO) consists of intermittently administering 100% oxygen at pressures greater than one atmosphere absolute (ATA) in a pressure vessel. This technology has been used to treat a variety of diseases. Positive effects are described particularly in patients suffering from chronic wounds or delayed- and non-unions after a fracture [15]. HBO has antimicrobial effects and accelerates fracture healing *in vitro* and *in vivo* [16]. Moreover, HBO reduces inflammatory response in an ischemic wound model [17]. Some recent animal studies suggested a beneficial effect of HBO in the treatment of bacterial infections of the bone marrow (osteomyelitis) [18–22]. In these studies, the effect of HBO therapy was more related to direct antibacterial activity of HBO than to a better phagocytosis of SA with rising intramedullary oxygen tensions during HBO therapy [17], [18]. Despite a great number of established animal models addressing bacterial bone infections, differences of pathophysiological mechanisms of bacterial infections of the bone marrow (osteomyelitis) and perioperative implant-associated infections (osteitis) are poorly understood. As osteomyelitis was induced by an intramedullary bacteria injection in the recent studies analyzing the effect of HBO, results from these studies can probably not be translated to implant-associated osteitis. Therefore, it remains unresolved whether HBO presents an immune- and fracture healing-modulating therapeutic approach for early implant-associated osteitis. A potentially beneficial effect of HBO therapy in implant-associated osteitis models might have a great impact on clinical practice. As osteitis animal models are considered to resemble the human situation, results might be translated “from bench to bedside” and HBO therapy might be an additive therapeutic option next to surgical and antibiotic therapy of osteitis.

The aim of the present study was to evaluate the effect of HBO on fracture healing, infection progress and immune response in an early implant-associated localized osteitis caused by SA in a murine femur fracture model.

## Material and methods

### Ethical statement

The present animal experiments were approved by the local institutional committee on animal care (“Landesamt für Naturschutz, Umwelt und Verbraucherschutz” of the federal state of North Rhine-Westphalia, Germany—file number: 87–5104.2010.A375) and are in line with the European Communities Council Directive (86/609/EEC). Specific effort was made to minimize the number of animals. Reporting of the results of the present study adheres to the “Animals in Research: Reporting in vivo Experiments”criteria (ARRIVE criteria) [23].

### Animals

Female wild-type BALB/c-mice were used for the study. The age ranged between 10 to 12 weeks with an average weight of 22 g. Mice were kept in the local animal research institution (animal facility of the Heinrich-Heine-University Düsseldorf, Zentrale Einrichtung für Tierforschung und wissenschaftliche Tierschutzaufgaben, ZETT, Germany) in standard polycarbonate (makrolon type II) cages under a conventional 12 h light–dark cycle (7:00 a.m. / p.m.). Mice had free access to food and water.

### The implant-associated osteitis model

A well-characterized and standardized implant-associated osteitis model in mice was used as described before [24,25]. In detail, mice were anesthetized by i.p. injection of xylazine (5 mg / kg body weight) and ketamine (100 mg / kg body weight). The thigh was gently shaved and cleaned with betadine and alcohol swabs. After a 2 cm skin incision along the left lateral thigh, the fascia was opened and the muscles were gently dissected to expose the femur. Afterwards, a 4-hole titanium locking plate with locking self-tapping micro-screws (MouseFix plate, RISystem, Davos, Switzerland) was applied to the femur. After plate fixation, an osteotomy using a Gigly saw (diam. 0.22 mm) was performed in midshaft of the femur. For the mice allocated to an osteitis group, implant-associated infection was induced by inoculation of the fracture gap with 1 μl of SA solution (strain ATCC 29213, averaged 1.94 x 10^3^ colony forming units / μl) [24]. All groups were re-anaesthetized 7 and 14 days after primary surgery and a standardized lavage with 250 μl phosphate buffered saline (PBS) twice and debridement of infected tissue was performed. Local surgical debridement was implemented with a sharp curette without involving the periosteum. The lavage fluid was recovered and PBS added to a total volume of 1 ml. The lavage fluid was further analyzed for the number of SA colony-forming units (CFU), polymorphonuclear neutrophils (PMN), Interleukin (IL) - 6, and the circulating free DNA (cfDNA). Parallel to the surgical lavage, blood serum was obtained from the tail vein on day 7, 14 and 28 and from heart puncture on day 56 for further analysis of serum bone healing markers: alkaline phosphatase (AP) and amino-terminal propeptide of type I collagen (PINP). Mice were euthanized by cervical dislocation on day 28 or 56.

### The hyperbaric oxygen therapy (HBO)

**Fig 1:**
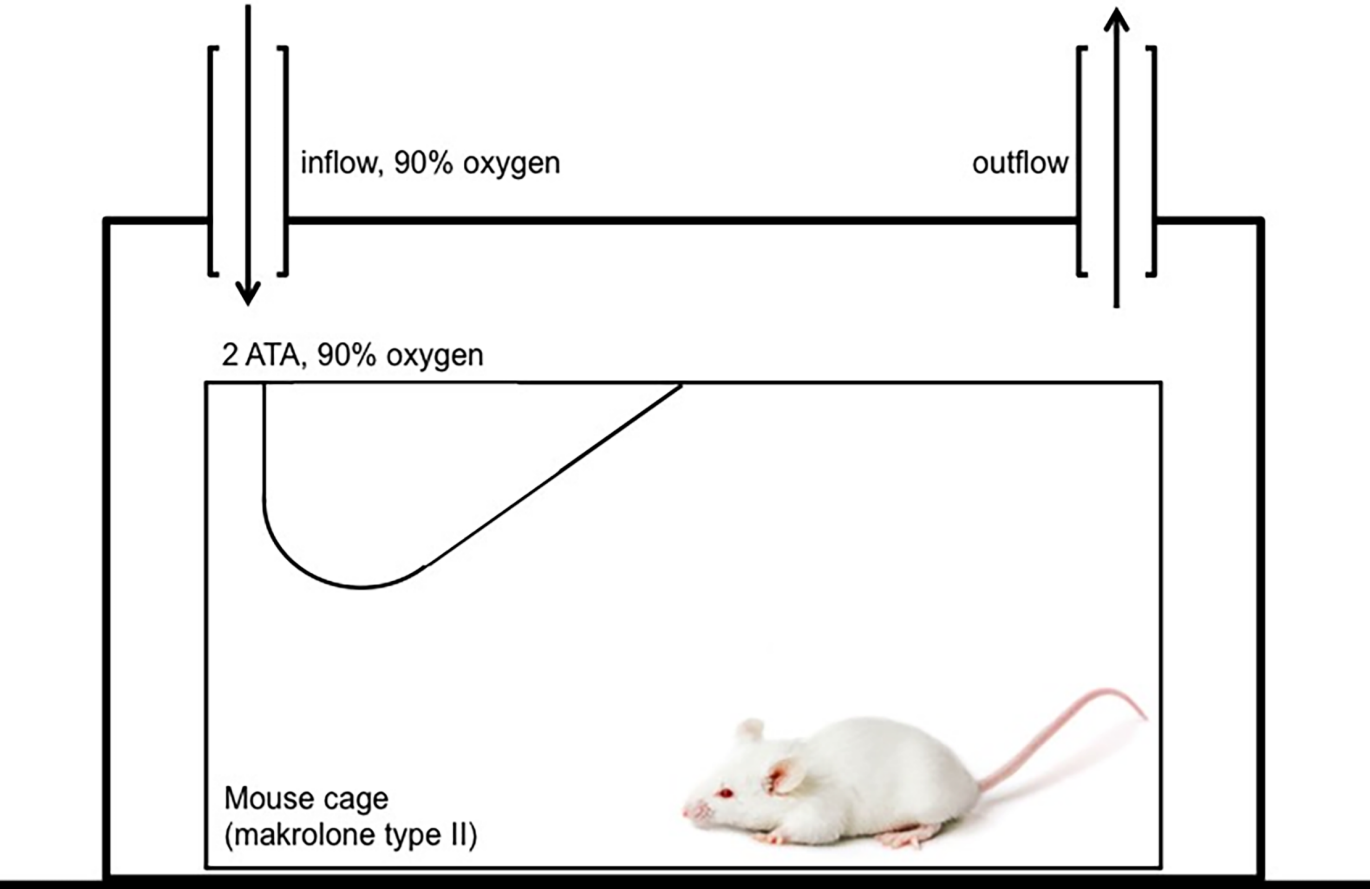
Setup for hyperbaric oxygen therapy in mice. Standard cages (Makrolon type II) with BALB/c mice were placed inside a closed chamber. 90% oxygen was applied over an in- and outflow for 2 minutes. Then mice were exposed to hyperbaric oxygen therapy for 90 Min at 2 absolute atmospheres (ATA).

The optimal setup for a HBO therapy in mice was evaluated in a series of pre-tests, as an HBO mouse model was not yet established. As a ventilation of 90% oxygen via air mask was not feasible in mice, the cages with mice allocated to the HBO group were put inside a closed chamber (Fig 1). First, 90% oxygen was applied over an in- and outflow for 2 minutes, and then mice were exposed to HBO 90 Min at 2 ATA. Mice were awake during the HBO therapy. Mice allocated to an HBO group received HBO therapy for 3 weeks (5 days per week) with the beginning on day 7.

### Allocating animals to experimental groups and sample size

In total, 120 BALB/c-mice were included in the experiments. To study physiological responses in reaction to an implant-associated infection and to evaluate fracture healing, infection recovery, and immune response after implant-associated infection of a femoral fracture in reaction to HBO therapy, mice were allocated to the following six experimental groups: In two sham-groups (12 mice / group) only a skin incision was performed. One of these sham-groups had HBO therapy as described above (90% oxygen, 2 ATA, 3 weeks). The other group had no further therapy (room air and conditions at all times). In two osteotomy groups one with and one without HBO therapy, osteotomy and plate osteosynthesis of the femur was performed (24 mice / group). In addition, infection was induced by inoculation of the fracture gap with SA in two osteitis groups one with and one without HBO therapy (24 mice / group).

### Experimental setup and outcome measures

Fracture healing was examined by x-ray analysis on day 0, 7, 28 and 56. Progress of infection in the wound was monitored by estimation of the counts of SA in lavage on day 7, 14, 28 and 56. The local inflammatory response was characterized by measuring the quantification of PMN, cfDNA and IL-6-levels in the lavage on day 7, 14, 28 and 56 after osteotomy. The serum AP and PINP were evaluated on day 7, 14, 28 and 56 in 12 mice per group.

### X-ray analysis

Standard anteroposterior radiographic images (MX20 Faxitron, Tucson, Arizona, USA; 40 kV, 16 mA) of the femora were taken under anesthesia on days 0, 7, 14, 28 and 56 after plate fixation. The fracture gap size was measured at the plate opposing cortical bone. The MouseFix plate has a length of 8 mm. This internal standard allows an exact calculation of the distance of the fracture gap. The fracture gap was classified by using a modification of a previously established score [25]: 1 point was considered a completely healed fracture gap. Decreasing diameters of fracture gaps representing fracture healing were rated with 2 points. A constant fracture gap meaning no healing was rated with 3 points, increasing facture gap was rated with 4 points, obvious osteolysis with 5 points and destruction of the femur with 6 points.

### Counts of colony-forming units (CFU)

The number of CFU was attained and determined from the lavage on day 7, 14 and 28 each. 200 ml lavage were serially diluted in PBS and four replicates of 10 μl of each dilution plated on Columbia Agar plates with 5% sheep blood and incubated under aerobic conditions at 37°C. Bacterial colonies were counted after 24 h. Results were specified as CFU per 1 ml.

#### Polymorphonuclear neutrophils (PMN)

The local inflammatory response was characterized by measuring the PMNs in the lavage using flow cytometry (FACSCanto II; BD Biosciences, Heidelberg, Germany) with the following antibody (FITC Rat Anti-Mouse Ly-6G; BD Pharmingen).

### Quantification of Interleukin (IL)-6

IL-6 levels in the lavage were determined using a commercially available IL-6 ELISA kit according to the manufacturer’s instructions (R&D Systems, Abingdon, UK). The lower detection limit for IL-6 was 16 pg / ml.

### Quantification of neutrophil extracellular traps (NETs)

NETs quantification in the lavage was performed by detecting cfDNA using the Quant-iT Pico green dsDNA assay (Invitrogen GmbH, Darmstadt, Germany). This assay is already used and described by our group [26,27]. The fluorescence intensity reflects the amount of DNA and was measured at excitation and emission wavelengths of 485 nm and 530 nm, respectively in a microplate reader (Victor3, PerkinElmer, Waltham, USA). A standard calibration curve by means of defined calf thymus DNA (Sigma, St. Louis, USA) amounts ranging from 0 to 2 μg / ml has been used in all analyses.

### Blood alkaline phosphatase levels (AP)

AP activity as a non-specific marker for bone healing was determined in serum. We used an AP Assay Kit measuring the AP activity directly without pretreatment (Abnova, Taipei, Taiwan). This method utilizes p-nitrophenyl phosphate that is hydrolyzed by ALP into a yellow colored product. The rate of the reaction is directly proportional to the enzyme activity and was measured at wavelengths of 405 nm 0 and 4 minutes after reaction (Victor3, PerkinElmer, Waltham, USA).

### Amino-terminal propeptide of type I collagen (PINP)

PINP concentration in serum was measured by a competitive ELISA assay for human N-terminal propeptide of collagen type I (Cloud-Clone Corp., Katy, USA). We followed the manufacture’s instruction while using this commercially available ELISA kit. The intensity of color was read in a microplate reader (Victor3, PerkinElmer, Waltham, USA) and is inversely proportional to the concentration of PINP in the sample.

### Statistical analysis

Statistical analysis was performed using GraphPad Prism5 (GraphPad Software, San Diego, CA). Real-valued data was first tested for normality using D´Agostino and Pearson normality test. If the variables themselves were normally distributed, the t-test was applied directly to the data to check whether the distribution means significantly differ. Not-normally distributed data was tested for statistical significance with two-tailed Mann–Whitney-test. P-values of 0.05 and below were considered significant. A trend towards significance was defined by a p-value between 0.1 and 0.05.

## Results

### Baseline data

Surgery was performed on 120 female wild-type BALB/c mice in the age range 10 – 12 weeks and a weight range of 18 – 27 g (mean 22 g). 18 mice died during the experimental procedures (overall mortality rate of 15%). There were no significant differences between the experimental groups. 102 mice were considered for further analysis.

### Fracture healing

**Fig 2:**
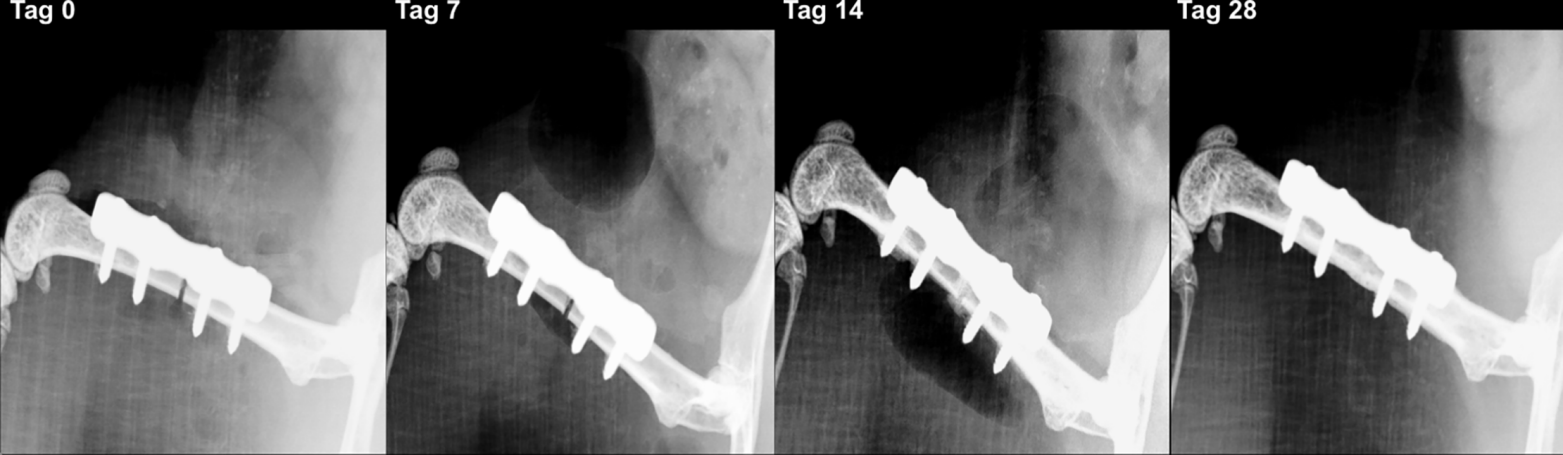
Radiographic analysis of fracture healing in non-infected mice. X-ray scans of the left femur in a mouse allocated to the osteotomy group after day 0, 7, 14 and 28. The fracture completely healed within the observation time.

**Fig 3:**
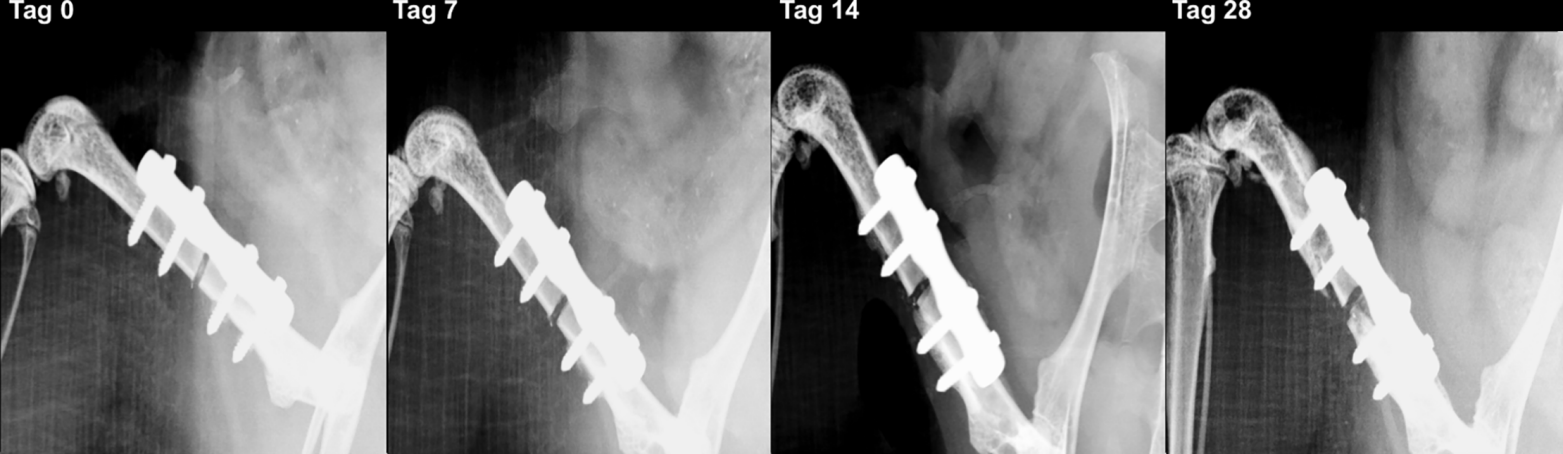
Radiographic analysis of fracture healing in infected mice. In contrast to the non-infected mice, mice allocated to both osteitis groups showed a higher frequency of nonunion. This mouse was allocated to the osteitis / HBO group and serial X-ray on day 0, 7, 14, and 28 shows development of a nonunion.

**Fig 4:**
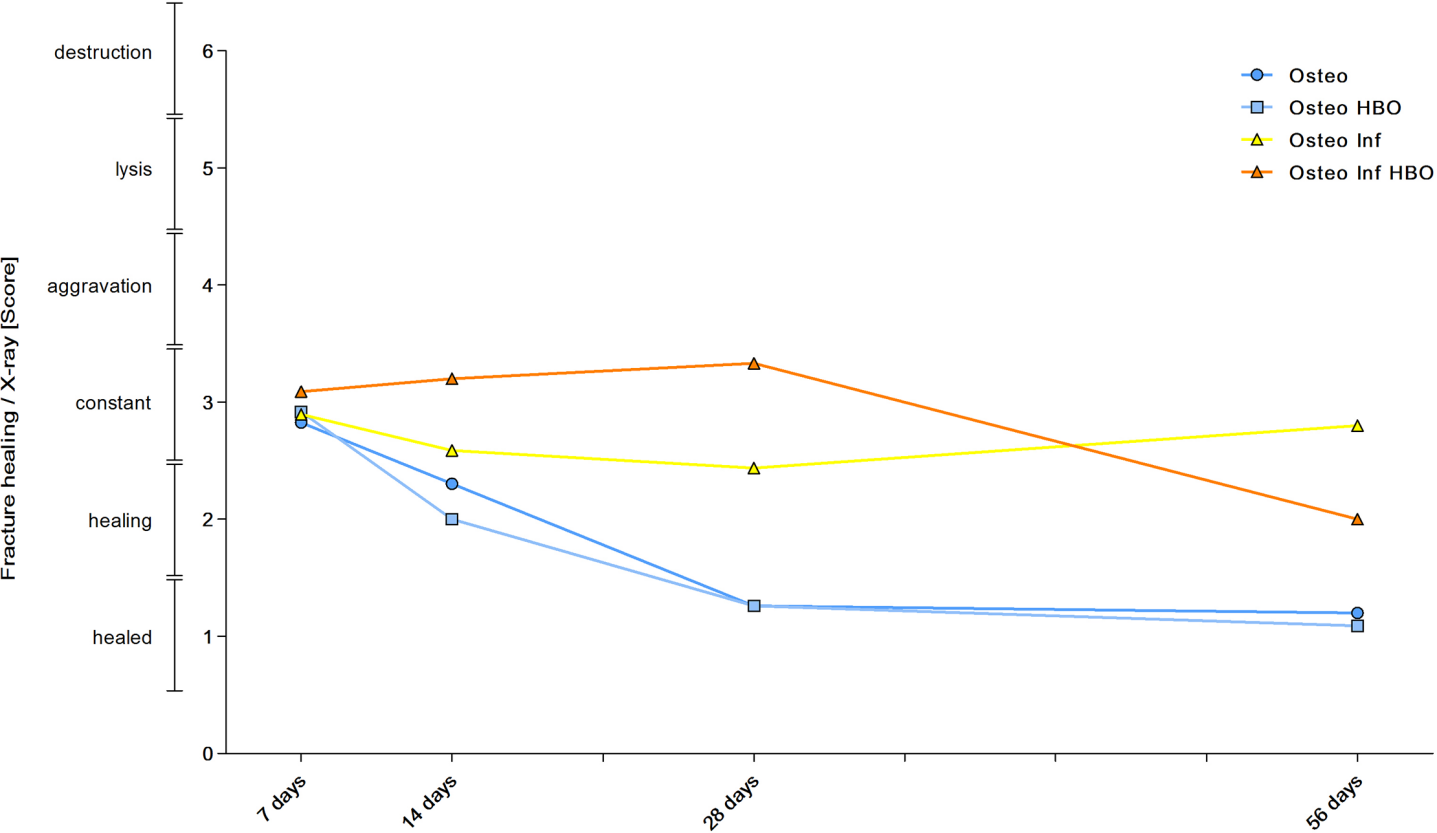
Mean score values of fracture healing. The fracture gap was measured on a.-p. X-ray scans and fracture healing was classified by a score. Individual and mean values of the score are summarized. Animals of the osteotomy and osteotomy / HBO group showed a sufficient healing of the fracture within the observation period and subsequently decreasing mean values as assessed by the score. Mean score values also decreased for the osteitis group over time but the score showed a greater individual heterogeneity and nonunion in individual animals. In contrast, median bone healing score increased for the osteitis HBO group till day 28 and improved on day 56.

**Fig 5:**
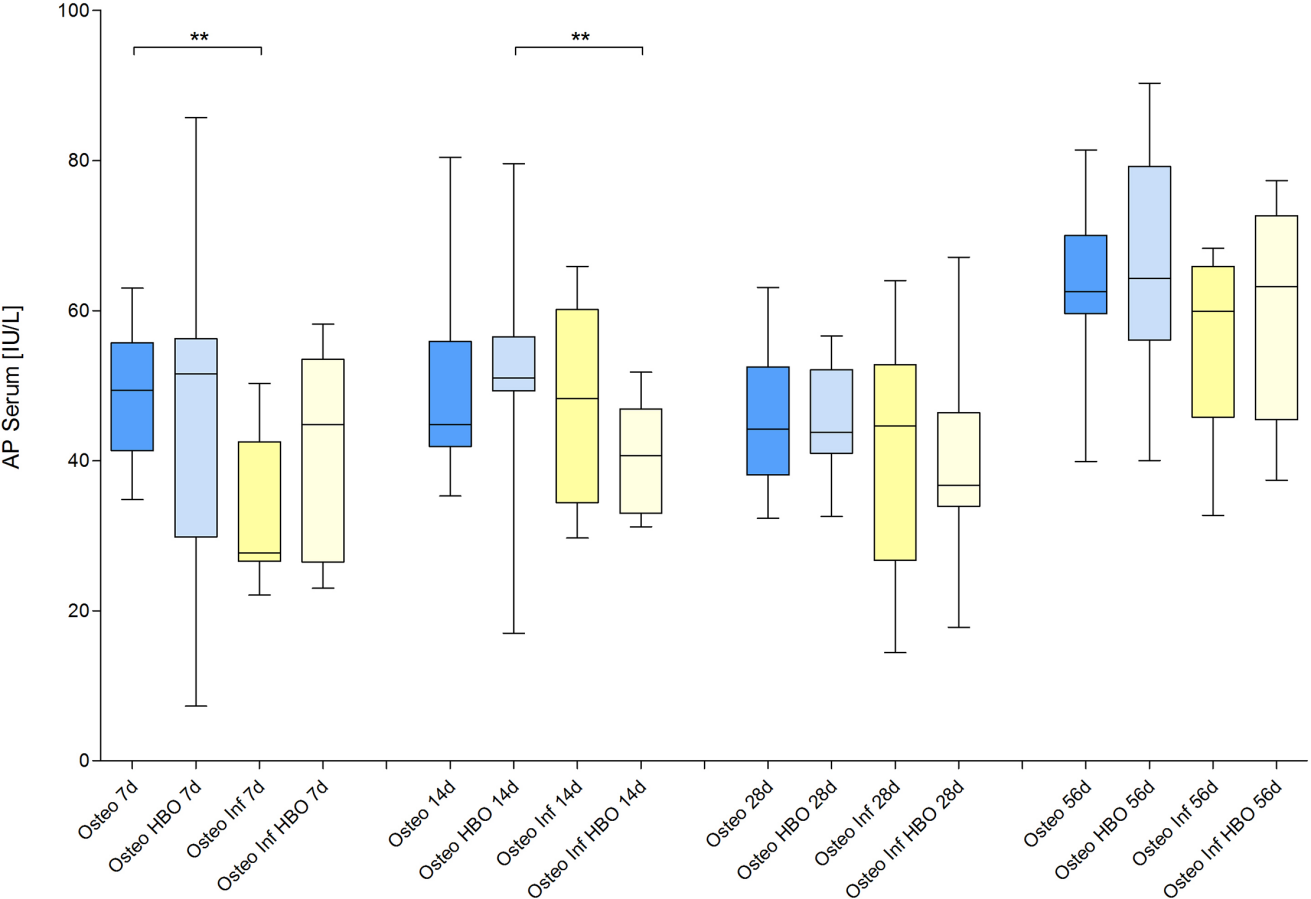
Blood alkaline phosphatase levels. Analysis of alkaline phosphatase (AP) in the blood serum revealed only significant differences between the osteotomy and the osteitis alone on day 7 and between osteotomy / HBO and the osteitis / HBO group on day 14.

**Fig 6:**
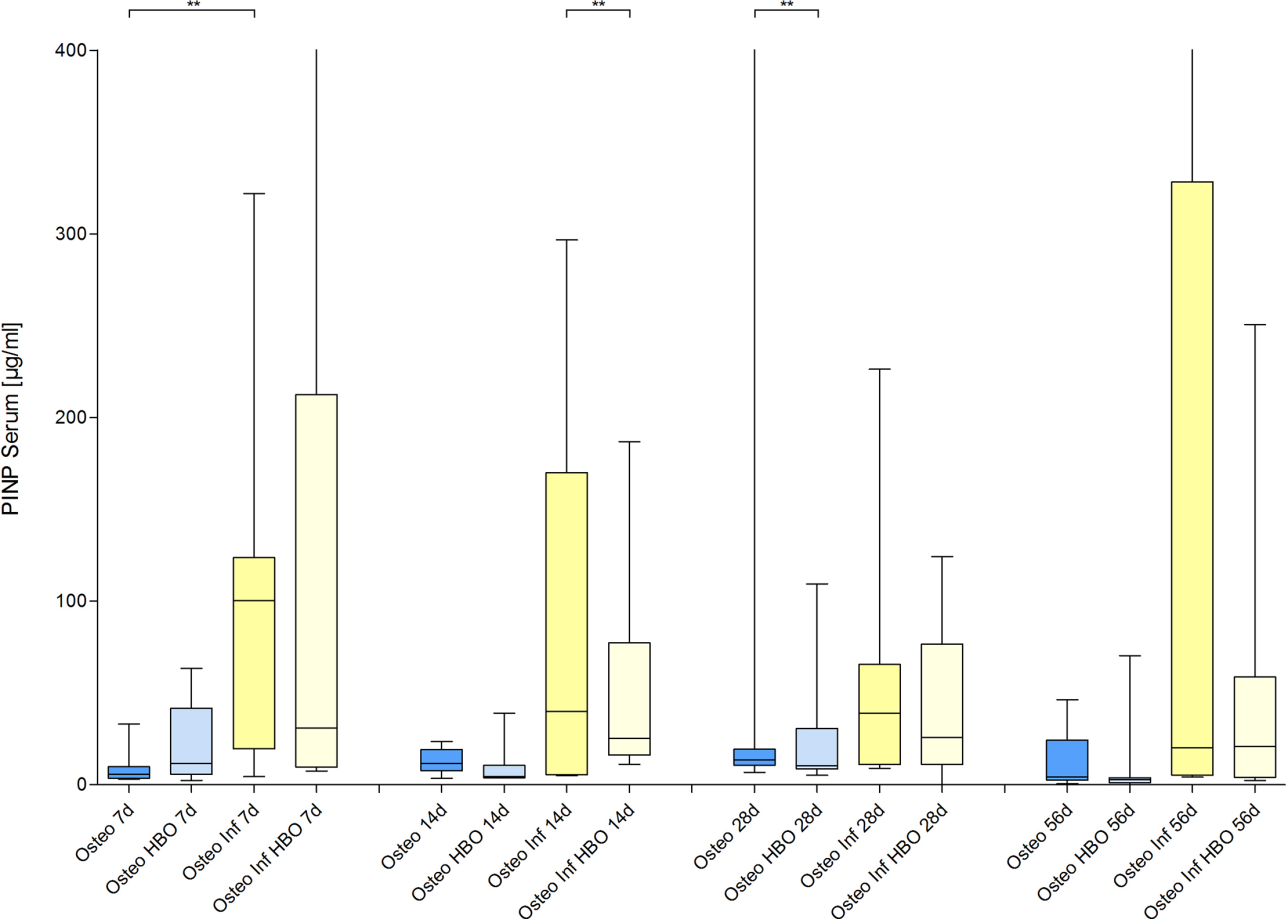
Blood amino-terminal propeptide of type I collagen levels. Analysis of amino-terminal propeptide of type I collagen (PINP) levels in blood samples revealed only a significant influence of HBO therapy after osteitis 14 days after infection.

On days 0, 7, 14, 28 and 56 after plate fixation anteroposterior radiographic images of the femora were taken under anesthesia. All mice with osteosynthesis without infection showed a healing fracture gap (Fig 2). In the animals of the osteotomy and osteotomy / HBO group, the fracture completely healed within the observation period. This was reflected by median bone healing score of 3 after 7 days, of 2 after 14 days and 1 after 4 weeks each. Mice with infection showed different results (Fig 3). Animals of the osteotomy / infection group had the same median values as the controls but showed a greater individual heterogeneity and nonunion in individual animals. In contrast, median bone healing score increased in the osteotomy / infection / HBO group till day 28. On day 56 after fracture, the mean score value in all experimental groups suggested a sufficient fracture healing in the majority of animals of all groups (Fig 4). Analysis of AP and PINP in the blood serum revealed only significant differences for both parameters between the osteotomy and the osteitis alone on day 7 and between osteotomy / HBO and the osteitis / HBO group on day 14 (Figs 5 and 6).

### Infection progress

**Fig 7:**
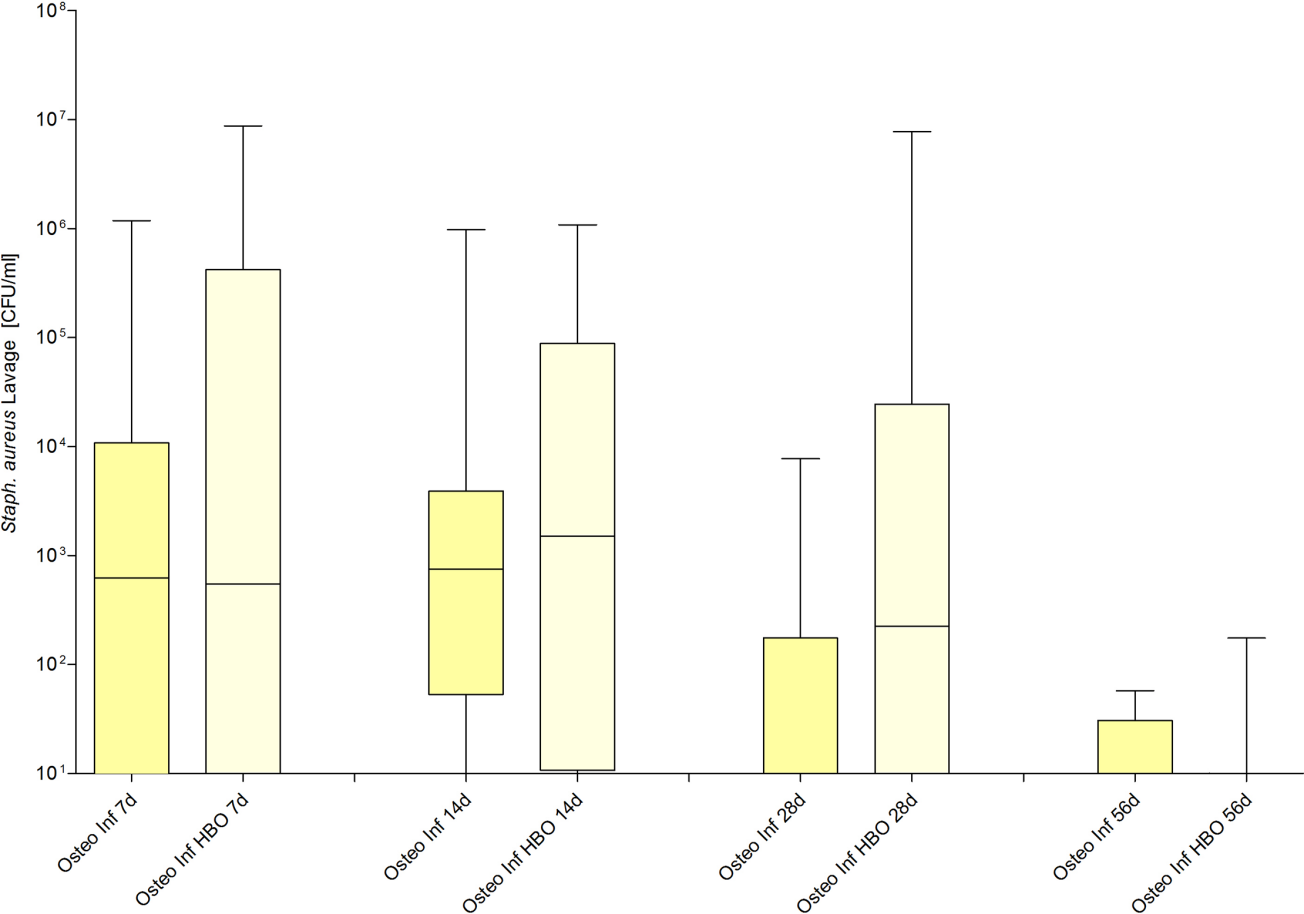
Detection of counts of Staphylococcus aureus (SA) around the facture side. Colony-forming units (CFU) in the lavage samples obtained on day 7, 14, 28 and 56.

Infection progress was verified by estimation of the numbers of CFU gained from the lavage on day 7, 14, 28 and 56. The sham-groups and the groups with osteosynthesis without infection showed no evidence for SA in all lavages (sham, sham / HBO, osteotomy and osteotomy / HBO: each 0 ± 0). In contrast, SA was verified in all infection groups. HBO therapy did not significantly influence SA CFU at any point in time (each p > 0.05, Fig 7).

### Inflammatory response

**Fig 8:**
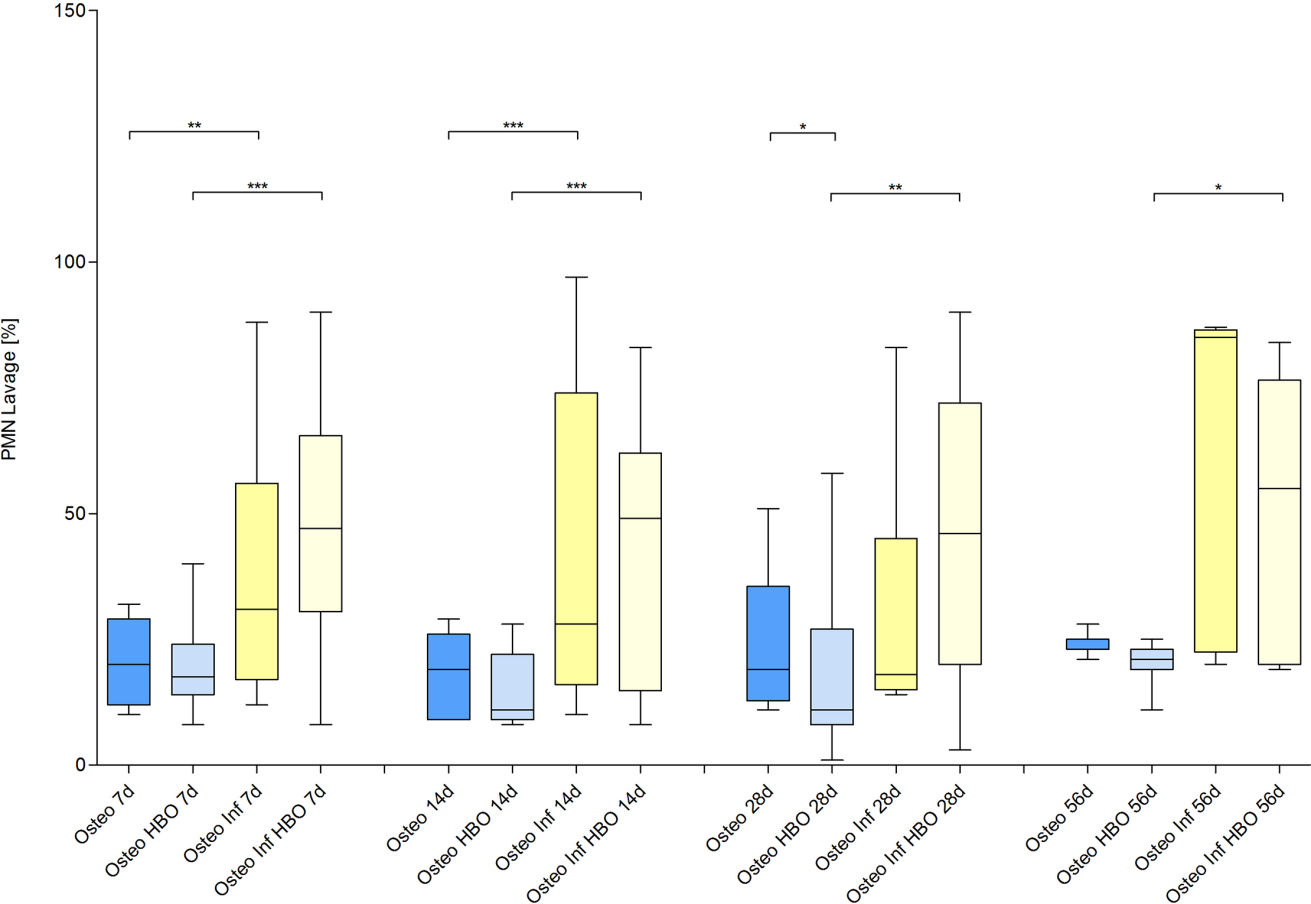
Quantification of polymorphonuclear neutrophils (PMNs).

**Fig 9:**
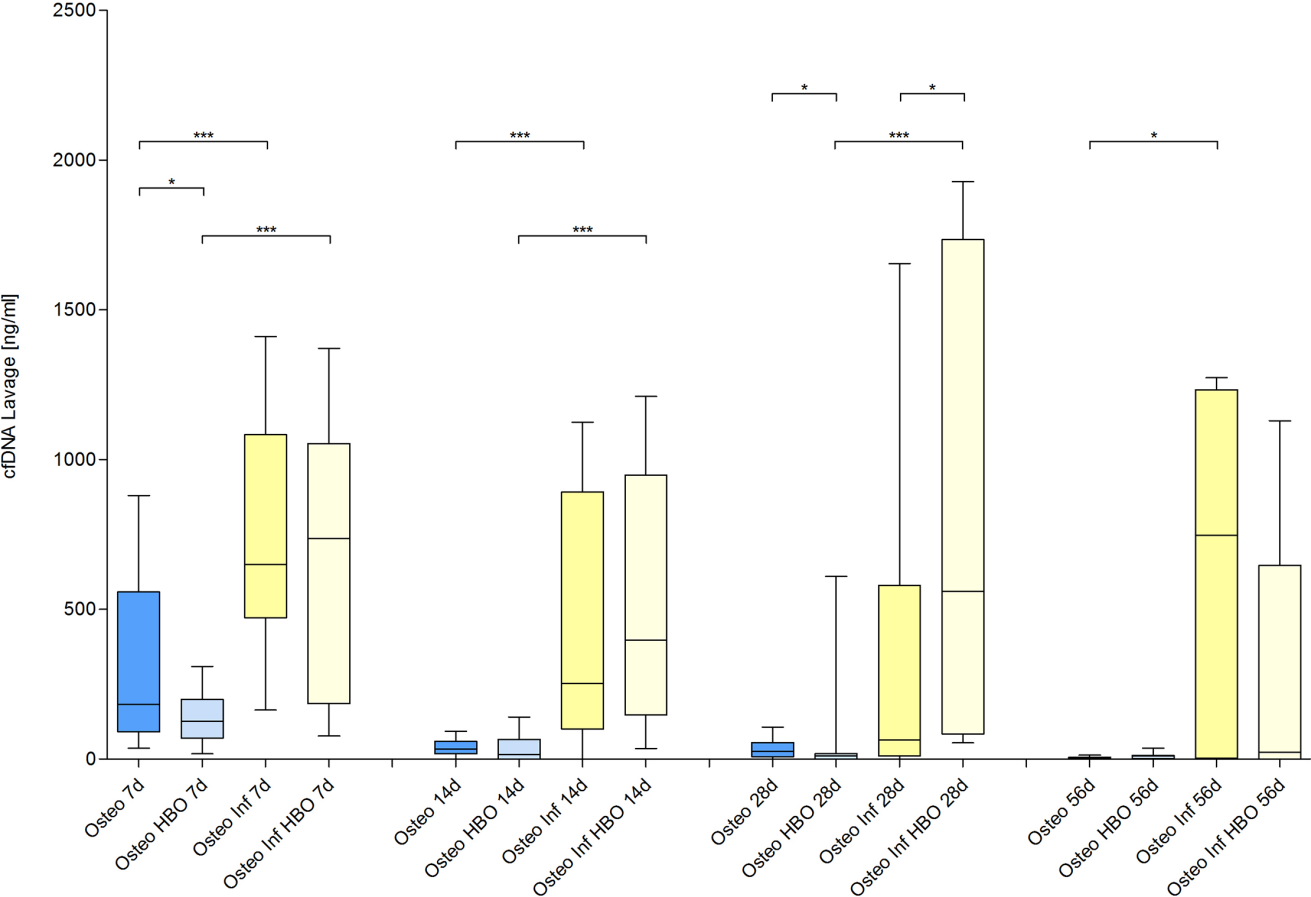
Quantification of circulating free DNA (cfDNA).

**Fig 10:**
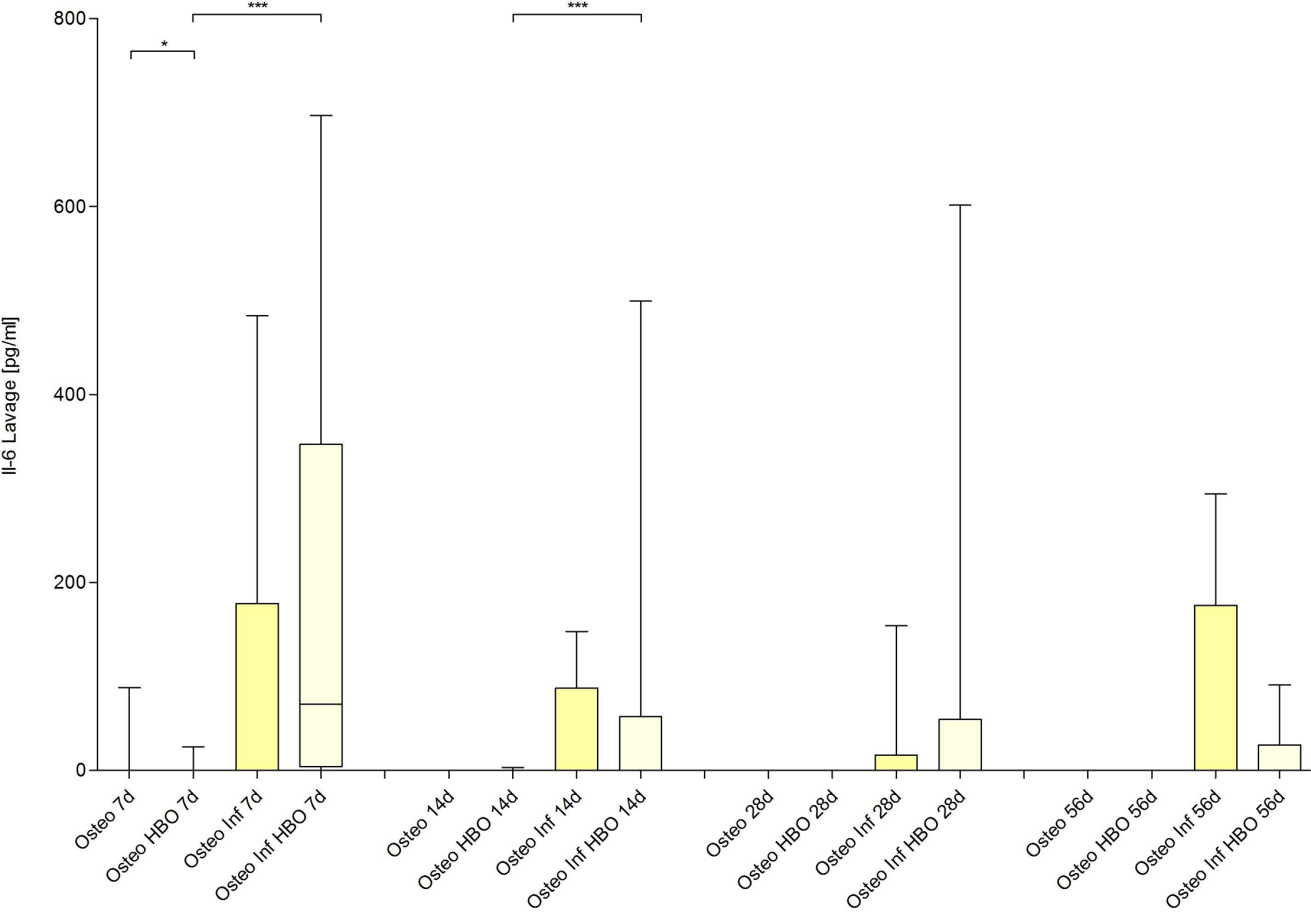
Quantification of Interleukin (IL)– 6 levels.

The inflammatory response was analyzed by quantification of the PMN, NETs and IL-6 (Figs 8, 9 and 10) levels in the lavage samples. A local infection induced a significant inflammatory response with an increase of local PMNs, NETs and IL-6 on day 7 and 14 (osteotomy vs. osteitis and osteotomy / HBO vs. osteitis / HBO), respectively. The osteitis / HBO but not the osteitis only group showed a significant increase of these outcome measures compared to the corresponding control groups on day 28 and 56. Next to infection, osteotomy alone, in comparison to the sham mice, results in an increase of proportional PMNs and NETs on day 7 and 14. HBO therapy after osteitis was associated with lower NETs levels on day 28 but did not have further significant influence on the following immune response during osteitis.

## Discussion

The main findings of this retrospective analysis of the present study evaluating the effect of HBO on fracture healing, infection progress and immune response in a mouse osteitis model is that HBO did not significantly influence bone healing and local infection in our osteitis mouse model and that mice exposed to the bone infection showed significant higher colony forming units and infection parameters.

HBO therapy was previously evaluated in several murine osteitis models: Hamblen induced osteomyelitis by injection of SA into the intramedullary cavity of rat tibia and described similar infection rates but an increased bone healing after established osteitis in the HBO groups as compared to the controls [18]. A similar model was used in rabbits: Here, HBO therapy led to a significant reduction of the intramedullary bacterial load but did not influence the radiographically assessed severity of the bone infection [20]. The therapeutic effectiveness of HBO therapy was more related to direct antibacterial activity of HBO than to a better phagocytosis of SA with rising intramedullary oxygen tensions during HBO therapy [19,20]. Recently, effects of HBO after induction of chronic osteomyelitis by intramedullary injection of SA into the right tibia were reevaluated in 6-month old Wistar rats: In comparison to the control groups, 2-weeks of HBO therapy led to a reduction of the mean SA CFU from mean 3.6 to 1.2 x 10^6^ CFU/g tibial bone and a 4-week HBO therapy led to a SA CFU reduction from 2.9 x 10^6^ to 6.2 x 10^5^ CFU/g tibial bone [22]. Accordingly, osteitis related radiographic changes improved as measured by a score [22]. However, it remains unclear if the improvements are significant. A later study of the group led to similar results for the HBO therapy alone and control groups [21].

In contrast to these results, the present results reveal neither a beneficial effect of HBO on bone healing nor on infection progress or inflammatory response in a mouse implant-associated osteitis model of the femur. This observation is in line with the two previous study evaluating. HBO therapy in a mouse osteitis model: Shandley and coworkers analyzed the impact of HBO in methicillin-resistant SA (MRSA), *Pseudomonas aeruginosa*, and *Klebsiella pneumonia* induced implant-associated osteitis of the tibia in C57BL/6 mice. In this model, HBO accelerated the growth of MRSA and resulted in more severe lesion scores [28]. Interestingly, only MRSA and *Pseudomonas aeruginosa* sufficiently induced a local osteitis but not *Klebsiella pneumonia*. The authors concluded, “HBO does not appear to be a useful treatment for osteitis in this model” [28]. More recently, additional effect of HBO combined with conventional antibiotic treatment (daptomycin and rifampicin) was compared to antibiotic standard regime in a mouse model of implant-associated osteomyelitis [29]. HBO (3 ATA, 100% for 60 min) was applied from day 11 to 14 after induction of osteitis by inoculation of a transcortical tibia implant with SA. In this setup, HBO therapy in combination with daptomycin and rifampicin did not significantly improve the cure rate or the bacterial load on the implants compared to antibiotic therapy alone. In contrast to our results, HBO induced a significantly elevated bone turnover as assessed by estimation of serum PINP and Tartrate-resistant acid phosphatase 5b concentration. Subsequently, the authors conclude that efficacy of antibiotic therapy cannot be improved by adjuvant HBO [29]. The effect of HBO was further evaluated in a different implant-associated model. Here, a Kirschner wire was introduced in the femoral cavity of Sprague-Dawley rats and osteitis induced by intramedullary injection of SA. The combination of vancomycin treatment and HBO therapy did not lead to a significant reduction of the bacterial load, the histo-pathologically evaluated degree of osteitis and IL-1b, IL-10, and TNF-a levels, in comparison to vancomycin treatment alone.

Therefore, in contrast to those studies, in which osteomyelitis was induced by intramedullary SA injection, models of implant-associated osteitis reveals no additional benefit of an HBO therapy. This observation might be due to several reasons or a combination of them: First, orthopedic implants might interfere with a potential effect of HBO therapy and might enable bacteria to escape from HBO-supported host defenses. Second, we performed stabilization of the femur with a plate and osteotomy. Injury of the bony cortex and the periosteum is therefore unique characteristic of our model and might be another explanation for the differences in regard to the previously mentioned injection models. Injury of the bony cortex and the periosteum might not be accessible for the benefits of HBO therapy. Again, Mader and coworkers attributes the therapeutic effectiveness of the HBO therapy not to a direct antibacterial activity but to a better phagocytosis of SA with rising intramedullary oxygen tensions during HBO therapy [19,20]. Rising intramedullary oxygen pressure was not evaluated in the present study, but elevation of the intramedullary oxygen tension might not necessarily promote phagocytosis in the fracture gap and the adjacent soft-tissue.

### Limitations

We acknowledge several limitations of our present study: First, in principle, the lack of influence of the HBO therapy on the osteitis might be due to a lack of induction of an infection constellation in the present model. However, we could rule this explanation out as our previous experiments and our controls clearly show that our model is sufficient to induce an osteitis constellation with a significant bacterial burden, an impairment of the natural bone healing and an inflammatory reaction. Second, not all possible parameters, such as intramedullary oxygen pressure or medullary perfusion, were analyzed in the present study. To minimize the number of experimental animals, the numbers of analysis are limited due to the small size of mice and researches have to focus on the most interesting parameters. Furthermore, we analyzed representative outcome measures for fracture healing, infection progress and immune response. Third, we cannot rule out that HBO might have an effect on other bacteria or poly-microbial infections. Especially poly-microbial infection and antibiotic resistance (in particular MRSA and multi-resistant *Enterobacteriaceae*) clinically might be problem as their rates increase [7,30]. Interestingly, Shandley and coworkers reported a HBO-mediated acceleration of the growth of MRSA in osteitis mice and subsequently an impaired bone healing. Fourth, other HBO protocols with variation of the HBO therapy, oxygen concentration and chamber pressure might lead to other results. Finally, our model might not necessarily resemble the human condition in every detail. Previously, it was questioned how far sepsis models resemble the human condition on a molecular level [31]. Again, our osteitis model shows a significant bacterial load, an impairment of the natural bone healing and an inflammatory reaction. These are important features of the human condition [7]. Finally, we used the term “osteitis” for description of the *SA* induced bone infection in the present model. A universally accepted definition of the terms “osteomyelitis” and “osteitis” is not yet established [2,3,32,33]. Especially in the Anglo-American literature, “osteomyelitis” is the preferred term to describe all kinds of bone infections [2,3]. In contrast, in particular in the German literature, the term “osteitis” is distinguished from the term “osteomyelitis”: First, different ways of infections were considered in osteitis and osteomyelitis [34]. In osteitis, way of infection occurs from outside to inside (centripetal way of infection) and in osteomyelitis way of infection appears from inside to outside (centrifugal way of infection). Infection progress in osteomyelitis was considered to be caused by hematogenous dissemination of pathogens and to firstly affect the bony marrow [34]. In contrast, in osteitis pathogens intrude from outside to inside in open fractures, peri-surgery and in our model. Second, the term “osteomyelitis” refers to infection of the bone mar row as the term “osteitis” is used to describe an involvement of the entire organ including the bone cortex [2,3]. In the present study, bacterial infection was induced after osteotomy of the femur. Therefore, the present model should resemble the situation of posttraumatic / postsurgical bacterial infections. As shown in the present and the previous studies, bacterial infection involves the bone marrow, the bone cortex and the surrounding tissue. Therefore, we used the term “osteitis”.

## Conclusion

The present osteitis model is sufficient to study fracture healing, infection progress and immune response following implant-associated SA-mediated osteitis in mice. However, HBO did not significantly influence bone healing and local infection in the present model.

## Supporting information

S1 Table Median values and standard deviation of all outcome measures. (XLSX)

S2 Table Significances of all outcome measures. (XLSX)
